# 

**Published:** 1895-01-26

**Authors:** 


					homas'5 Hospital
.ascoit Western Infirmaax
We hospital.
PRICE TWOPENCE. p&k
No. 435, Vol. XVII,?Jan. 26th, 1895. b 1
rRegistered at the General Post Office cs a Newspaper,
Jan. 26, 1895.
WITH EXTRA NURSING SUPPLEMENT.
Per Annum, 10s. 6d., post free.
Monthly Parts, 6d.; post free, 8cL
Ditto per Annum, 8s. 6d? post free.
No. iSS. Vol. XVII.
WITH EXTRA NURSING SUPPLEMENT.
'RWICH HOSPI TAL
Saturday, Jan. 26th, 1895.

				

